# Optimization of Preparation Conditions of Poly(m-phenylene isophthalamide) PMIA Hollow Fiber Nanofiltration Membranes for Dye/Salt Wastewater Treatment

**DOI:** 10.3390/membranes12121258

**Published:** 2022-12-13

**Authors:** Qinliang Jiang, Kaisong Zhang

**Affiliations:** 1 Institute of Energy Research, Jiangxi Academy of Sciences, Nanchang 330096, China; 2 Key Laboratory of Urban Pollutant Conversion, Institute of Urban Environment, Chinese Academy of Sciences, Xiamen 361021, China; 3 Key Lab of Marine Environment and Ecology, Ministry of Education, Ocean University of China, Qingdao 266100, China

**Keywords:** hollow fiber, nanofiltration, PMIA, preparation conditions, dye/salt wastewater

## Abstract

Externally selective thin film composite (TFC) hollow fiber (HF) nanofiltration membranes (NFMs) hold great industrial application prospects because of their high surface area module. However, the complicated preparation process of the membrane has hindered its mass manufacture and application. In this work, PMIA TFC HF NFMs were successfully prepared by the interfacial polymerization (IP) of piperazine (PIP) with 1,3,5-benzenetricarbonyl trichloride (TMC). The effect of the membrane preparation conditions on their separation performance was systematically investigated. The characterized results showed the successful formation of a polyamide (PA) separation layer on PMIA HF substrates by the IP process. The as-prepared HF NFMs’ performance under optimized conditions achieved the highest pure water permeability (18.20 L·m^−2^·h^−1^, 0.35 MPa) and superior salt rejection in the order: R_Na_2_SO_4__ (98.30%) > R_MgSO_4__ (94.60%) > R_MgCl_2__ (61.48%) > R_NaCl_ (19.24%). In addition, the as-prepared PMIA HF TFC NFMs exhibited desirable pressure resistance at various operating bars and Na_2_SO_4_ feed concentrations. Excellent separation performance of chromotrope 2B dye was also achieved. The as-prepared PMIA HF NFMs thus show great promise for printing and dyeing wastewater treatment.

## 1. Introduction

In the textile printing industry, the production process inevitably generates and discharges large amounts of wastewater with high salinity and high color [1,2]. Unfortunately, a large number of synthetic dyes are present in this wastewater that are difficult to degrade because of their stable chemical structure and high molecular weight. Consequently, excessive discharge of this wastewater causes serious pollution to the water ecological environment and wastes useful and economical resources [3,4]. In some fields of application, dyes are of great economic importance, which has therefore necessitated their recycling from dyeing wastewater. In addition, a large number of inorganic salts (such as ~6% NaCl or ~5.6% Na_2_SO_4_) in the wastewater also give the printing and dyeing wastewater a massive potential recycling value [5]. At present, the processes of separating valuable dyes and inorganic salts contained in printing and dyeing wastewater (including dye purification, inorganic salt desalting, and treated wastewater reuse) have been of global concern [6]. Chiefly, this category of wastewater has been treated by flocculation, coagulation, oxidation, and biological processes [7,8]. Unfortunately, most of these treatment methods suffer the same defects, high energy consumption and cost, and the valuable resources contained in these wastewaters cannot be fully recycled [9].

Compared with traditional wastewater treatment technology, the NF separation process has the advantages of low investment, easy operation, high dye recovery rate, and no adverse impact on the environment, etc. [10,11,12]. At present, NF components are mainly coil membrane components which have the merits of high filling density and low cost of membrane components [13]. However, their disadvantages include increased pressure drop caused by isolators in fluid channels, susceptibility to contamination, and failure to backflush. As a result, they usually require a lot of pre-processing. On the contrary, hollow fiber membranes have a higher packing density than flat sheet membranes and have backwashing potential in fouling layer removal. At the same time, the usual upstream pretreatment is omitted, thereby resulting in higher process efficiency and lower operation costs [14]. Nevertheless, the commercial preparation technology of hollow fiber nanofiltration membranes is not completely mature, and there are not many mature and stable hollow fiber nanofiltration membranes in the business. Therefore, adopting more simple and mature membrane preparation parameters is pertinent to preparing economically advanced nanofiltration membranes.

The most common commercial TFC NFMs currently directed at treating dye wastewater treatment are usually prepared by the IP method [15,16]. Currently, the TFC NFM available in the exchanges is mainly composed of three layers: the non-woven support layer, a porous polymer substrate layer, and an ultra-thin barrier layer [17]. Meanwhile, HF TFC NFM is a self-supporting structure, which is mainly composed of two parts: a porous polymer substrate, and dense layers [18]. Although the NFM’s performance mainly depends on the structure of the dense layer, the material properties of the polymer substrate layer also have an obvious effect on the microstructure of the membrane separation layer [19,20]. Currently, most HF TFC NFMs use polysulfone (PSF), polyethersulfone (PES), or polyvinyl chloride (PVC) ultrafiltration membranes as substrates because of their attractive physical and chemical properties, which facilitate IP [21,22,23]. However, the low chemical resistance of these substrates to ketones and alcohols limits NFMs’ structural stability. Given this, researchers have attempted to develop different kinds of porous support layers, such as polypropylene (PP), polyethylene (PE), and polyvinylidene fluoride (PVDF) microfiltration membranes, which are used to produce commercial non-polar polymer membranes because of their high solvent stability, good pressure resistance, low cost, and high porosity [23,24]. However, their poor surface wettability restricts the aqueous solution’s spread, making it difficult to generate a polyamide separable layer on the substrate surface by the IP process. Therefore, this justifies the need to find new substrate materials to overcome the above limitations.

Poly (m-phenyleneisophthalamide) (PMIA) has been reported to be a very attractive material for membrane preparation [25], because of its outstanding chemical stability, lasting thermal stability, excellent flame retardance, relatively low cost, easy processing, and excellent mechanical properties [26,27,28]. For instance, Chen et al. prepared a thermally stable PMIA TFC NFM by the IP method on PMIA flat substrates [27]. However, the preparation process of flat sheet TFC NF membranes is relatively simple and mature, and the prepared membranes do not have significant separation advantages compared to other reported related NF membranes. Additionally, Wang et al. prepared a PMIA HF substrate and successfully synthesized a polyamide separation layer on the substrate by the IP method. The prepared membrane displayed relatively good separation performance [24]. However, this study only reported the separation performance of the NF membrane under one preparation condition. As a matter of importance, the IP parameters usually have a great influence on the performance of HF TFC NFMs during the process of large-scale industrial production. Therefore, effective optimization of the process parameters in membrane preparation is of great importance.

On this backdrop, piperazine (PIP) (selected as the monomer of the water phase) and 1,3,5-benzenetricarbonyl trichloride (TMC) (selected as the monomer of the organic phase) were employed to develop a PA separation layer on the outer surface of a PMIA HF substrate by the IP process. The preparation conditions of the HF NFMs were thoroughly investigated by optimizing the membrane preparation process (including the effects of monomer concentration, water contact time, and IP time) on the properties of the membranes. The separation characteristics of prepared membranes under different operating conditions were evaluated from two aspects of permeability and rejection rate using a simulated saline dye solution, and the optimal preparation conditions were obtained.

## 2. Experimental

### 2.1. Materials

**Figure 1:**
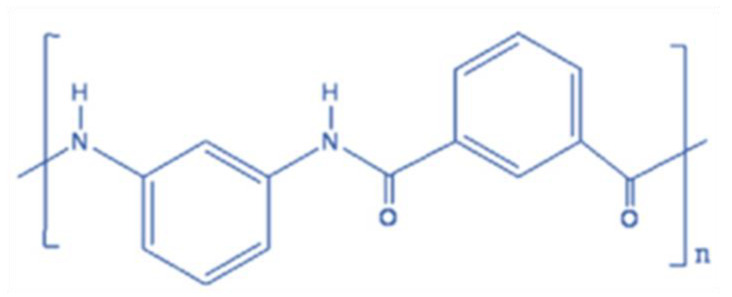
Chemical structure of PMIA.

Hollow fiber substrates were prepared using PMIA (DuPont Company, Wilmington, DE, USA). LiCl was acquired from Sinopharm Chemical Reagent Co., Ltd. (Shanghai, China). Polyethylene glycols (PEG, Mw = 400 g mol^−1^), PIP, TMC, and four inorganic salts (Na_2_SO_4_, MgSO_4_, MgCl_2_, NaCl) were procured from Shanghai Aladdin Reagent Co. Ltd. (Shanghai, China). N,N-dimethylacetamide (DMAC, >99%) was purchased from Shanghai Jingwei Chemical Co., Ltd. (Shanghai, China). Chromotrope 2B (Mw = 513 g mol^−1^) and Janus Green B (511 g mol^−1^) were acquired from Sigma Aldrich (Darmstadt, Germany) and the characteristics of these two dyes are detailed in Table 1. All other chemicals involved in experiments were acquired from Sinopharm Chemical Reagent Shanghai Co. (Shanghai, China). The chemical structure of PMIA were shown in Figure 1.

### 2.2. Preparation of PMIA Substrate and HF NFMs

#### 2.2.1. Preparation of PMIA HF Substrate

The PMIA HF substrate was fabricated using dry–wet phase inversion technology [29]. The casting solution formulation of the above substrate was 14 wt.% PMIA, 4 wt.% LiCl, 3 wt.% PEG, and 79 wt.% DMAC. The pre-determined PMIA, solvent, and additives were dissolved in the flask in an appropriate order and pre-dissolved at 70 °C for a certain time, followed by mechanical continuous stirring for at least 12 h. After the polymer was completely dissolved, the dope solution was defoamed for use with continuous stirring for at least 12 h. After the PMIA fibers were completely dissolved, the casting solution was vacuum defoamed for use. The polymer solution and bore fluid are extruded together under the action of a precision syringe pump equipped with a device to control the flow rate of the viscous fluid. The nascent fibers were collected by the roller with a fixed take-up speed through an air bath and a coagulant bath (tap water), and the related performance parameters of the PMIA HF substrates are detailed shown in Tables S1 and S2. The detailed spinning parameters are summarized in our previous study [30], and the casting equipment was shown in Figure S1. The picture and SEM cross-section images of the PMIA HF substrate was shown in Figure S2.

#### 2.2.2. Preparation of PMIA HF TFC NF Membranes

The PMIA HF NFMs were obtained using IP technology. First, the homemade PMIA HF porous substrates were soaked in deionized water to be used, drained in air, and then put into the aqueous phase solution containing PIP to be submerged for a few minutes. Then the PMIA substrate was removed from the aqueous phase, and the excess droplets on the surface of the substrate were gently absorbed with paper towels and then dried with a hair dryer for a few minutes. Next, the blown dry PMIA substrate was quickly transferred to the oil phase solution of TMC for interfacial polymerization reaction for a few seconds. Finally, the membrane was removed and dried in an oven at 60 °C for 2 min and stored in deionized water until use before testing.

#### 2.2.3. Characterizations of Membranes

SEM images of the prepared PMIA HF substrates and PMIA HF NFMs were collected by a field emission scanning electron microscope (FE-SEM, HITACHI S4800, Hitachi, Tokyo, Japan). A Hitachi E-1010 ion sputtering coating machine was used to sputter gold nanoparticles in a vacuum before testing. Fourier transform infrared spectroscopy (FTIR, Nicolet iS50, Nicolet, Madison, WI, USA) with a wavenumber range of 650–4000 cm^−1^ was applied to analyze the IP reaction occurring on the PMIA HF substrates. The surface elements of the relevant membrane were determined by X-ray photoelectron spectrometry (XPS, Axis Supra, Kratos, Manchester, UK). The water contact angle (CA) of the prepared membranes was evaluated by a CA analyzer (DSA100, KRUSS, Hamburg, Germany). The surface roughness of the membranes was analyzed by atomic force microscopy (AFM, Dimension Icon, Bruker, USA). The surface zeta potential of the membranes was tested using streaming potential measurements with a SurPASS electrokinetic analyzer (SurPASS 3, Anton Parr GmbH, Glaz, Austria).

#### 2.2.4. Permeation Experiments

The permeability of the prepared HF NFMs was investigated by a crossflow filtration experiment after the pre-pressured process at 0.5 MPa with deionized water for 5 h and collected permeate at 0.35 MPa. The effective membrane area was about 14.1 cm^2^. The permeability of the membrane was then characterized by the rejection of the Na_2_SO_4_ aqueous solution (2000 ppm). The conductivity of inorganic salts was tested with a conductivity meter (SensION + EC5, HACH, Loveland, CO, USA). To further evaluate the treatment potential of the membranes in question for salt-containing dye solutions, they were used to separate simulated textile wastewater. The concentration of dyes in the produced water is determined by a UV-Vis spectrophotometer (UV3600, Shimadzu, Kyoto, Japan). Permeability flux (J) and rejection (R) were expressed by Equations (1) and (2), respectively.

(1) $$J_{w}=\frac{Q}{A\cdot t}$$

(2) $$R=\left(1-\frac{C_{p}}{C_{f}}\right)$$

where *J_w_* is the permeation flux of the PMIA HF TFC NF membrane (L·m^−2^·h^−1^), *Q* is water volume (L), *A* denotes the membrane area (m^2^), and t is the filtration time (h). R is solute rejection, *C_f_* is feed concentration (mg/L), *Cp* is permeate concentration (mg/L).

## 3. Results and Discussion

### 3.1. The Effects of Preparation Conditions

To obtain optimal preparation conditions, the effects of IP reaction conditions (PIP and TMC concentration, aqueous phase immersion time, IP reaction time) on the membrane properties of the PMIA HF NFMs were investigated separately.

**Figure 2:**
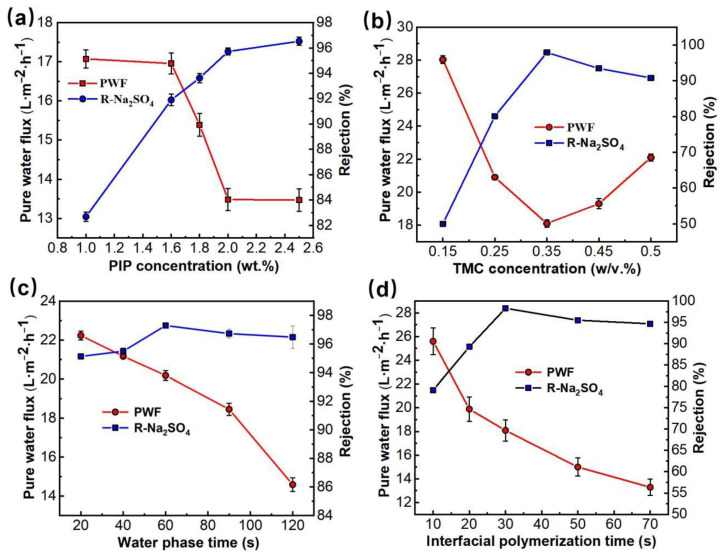
The effects of preparation conditions on the separation properties of the PMIA HF TFC NF membrane. (**a**) the effect of PIP concentration (TMC concentration was 0.35 *w*/*v*%, and the water phase soak and IP reaction time were 1 min and 20 s, respectively). (**b**) the effect of TMC concentration (PIP concentration was 1.8 wt.%, and the water phase soak and IP reaction time were 1 min and 20 s, respectively). (**c**) the effect of water phase soak time (PIP and TMC concentrations were 1.8 wt.% and 0.35 *w*/*v*%, respectively, and the IP reaction time was 20 s). (**d**) the effect of IP reaction time (PIP and TMC concentrations were 1.8 wt.% and 0.35 *w*/*v*%, respectively, and water phase soak time was 1 min).

First, the effects of PIP in the aqueous phase solution and TMC concentration in the oil phase solution on the membrane separation performance of PMIA HF NFMs were investigated. The nanofiltration test conditions were 25 °C and 0.35 MPa, respectively. The results were presented in Figure 2a,b. Figure 2a illustrated the curve of PMIA HF NFMs’ permeability with the change of water phase PIP concentration. As seen in Figure 2a, as the PIP concentration in the aqueous phase was enhanced from 1.0 wt.% to 2.5 wt.%, the pure water flux gradually decreased from 17.1 L·m^−1^·h^−1^ to 13.5 L·m^−1^·h^−1^, whereas the Na_2_SO_4_ rejection increased from 82.6% to 96.5%. However, when the PIP concentration was less than 2.0 wt.%, the PWF decreased rapidly and the Na_2_SO_4_ rejection rate rose quickly. With the PIP content exceeding 2.0 wt.%, both the PWF and the membrane rejection performance changed slowly. This is mainly because when the PIP concentration is less than 2.0 wt.%, with the rise of the PIP concentration, TMC and PIP undergo a polymerization reaction at the interface to form a dense network macromolecular layer, resulting in a rapid decreased in membrane flux and a rapid increased in salt rejection. When the PIP concentration is greater than 2.0 wt.%, the amount of PIP diffusion into the oil phase increases, resulting in a gradual thickening of the polyamide layer thickness and only a slight increase in rejection rate.

The influence of TMC concentration in the oil phase solution on membrane permeability is displayed in Figure 2b. When the concentration of TMC was lower than 0.35 *w*/*v*%, the effects of TMC and PIP concentrations on the performance of PMIA HF NFMs showed the same trend. When TMC concentration is further increased (*w*/*v*% > 0.35), it is mainly due to the reaction rate being too fast, which resulted in an incomplete reaction of the PA layer. As a result, the flux of the membrane increased and salt rejection declined.

Figure 2c showed the variation curve of PMIA HF TFC NFMs’ performance with the water phase soak time. When the immersion time of the aqueous phase was less than 60 s, with the prolonging of water phase soak time, the water flux of the membrane showed a downward trend, while the salt rejection showed an upward trend. This is mainly because the amount of PIP monomers in the permeating membrane hole will gradually increase with soaking time in the aqueous phase. The density of the effective separation layer thickness formed on the surface of the basement membrane rose continuously. Thus, the NFMs flux decreased and the rejection rate increased.

Figure 2d shows that with the IP reaction time being enhanced from 10 to 30 s, the PWF decreased while the Na_2_SO_4_ rejection rate rose quickly. This is mainly because PIP and TMC were highly reactive monomers, and the contact reaction rate constant between them was very fast, leading to a rapid interfacial polymerization reaction at the interface between the two phases, resulting in a dense separation layer, which leads to a decrease in flux and a rise in rejection rate. While the IP reaction time exceeded 30 s, the Na_2_SO_4_ rejection tended to stabilize and the permeation flux decreased.

In summary, the optimal preparation conditions by combining the above membrane production processes parameters of the prepared PMIA HF TFC NF membrane were achieved at an aqueous phase PIP concentration of 1.8 wt.%, aqueous phase soak time of 1 min, oil-phase TMC concentration of 0.35 *w*/*v*%, and IP time of 30 s.

### 3.2. Membrane Characterization

#### 3.2.1. Surface Properties

**Figure 3:**
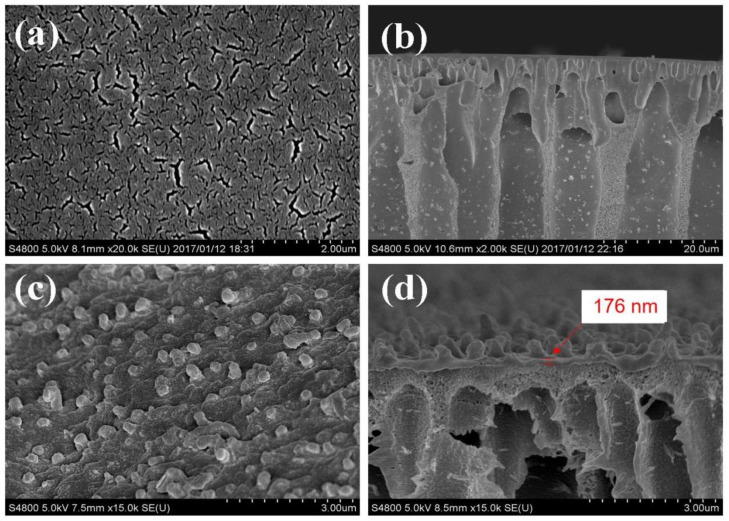
Morphologies of surface (**a**,**c**) and cross-section (**b**,**d**) of PMIA hollow fiber substrate membrane and NF membrane, respectively.

The SEM images of the PMIA substrate and the PMIA HF TFC NFMs are depicted in Figure 3. Figure 3a shows that the surface of the PMIA substrate is smooth and porous, while its cross-section in Figure 3c reveals a defect-free sub-structure and other multiples of cross-section information are detailed in Figure S2. As can be seen from Figure 3b, there are no visible pores on the surface of the PMIA HF NFMs. On the contrary, compared to the morphologies of the PMIA substrate surface, there are some rounded bumps on the surface of the PMIA NFM. Furthermore, the cross-section of the PMIA HF NFMs shows that a dense layer with a thickness of about 176 nm was formed on the surface of the PMIA substrate. In Figure 3d, there was an indication that the PA layer was resoundingly developed on the surface of the PMIA HF substrate.

**Figure 4:**
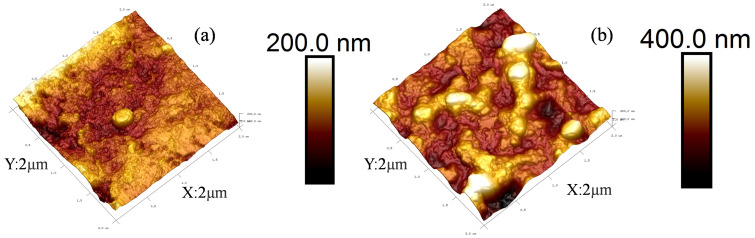
AFM photos of PMIA substrate (**a**) and PMIA HF TFC NF membrane (**b**).

The AFM photos of the PMIA substrates and PMIA HF TFC NFMs were shown in Figure 4. It can be seen from Figure 4b that, compared with Figure 4a, the PMIA NF membrane surface presents a ridge, and valley structure, which is consistent with SEM photos of the PMIA HF NFM’s surface. This is a typical reactive film layer formed mainly due to the polymerization of PIP in the aqueous phase and TMC in the oil phase when the aqueous and oil phases come into contact [27,31]. In addition, as can be seen from the data in Table 2, as a result of the ridge and valley structure of the polyamide layer, the Ra value of the PMIA NF membrane surface is greater than the Ra value of the substrate, which means that the roughness of the PMIA NF film surface is higher than the roughness of the substrate surface. Therefore, the results further reinforced the fact that a dense PA layer was triumphantly formed on PMIA HF multi-aperture support layer.

**Figure 5:**
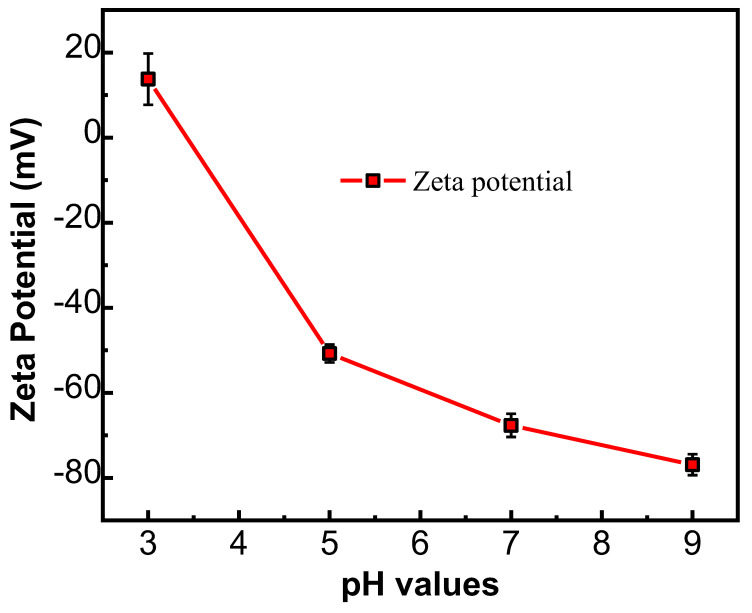
Potential values of PMIA HF TFC NF membrane at various pH values.

The Zeta potential of the NFM’s surface presents different surface charge properties at different pH values of the test solution. Figure 5 reveals the Zeta potential of the PMIA HF NFMs at different pH values. Under low pH conditions, due to the adsorption of H^+^, the NF membrane surface has a positive charge. At a high pH value, as a result of OH^−^ adsorption, the membrane surface possesses a negative charge [32]. The PMIA HF NFM’s surface presented a negatively charged property when the pH values were between 3 and 9. This is mainly related to the -COOH generated by the hydrolysis of TMC with an incomplete IP reaction [33]. In addition, to further understand the variation of hydrophilicity between the PMIA HF substrate and PMIA HF NFM’s surface, the water CA values of the PMIA HF support layer and PMIA HF NFM’s surface were tested, and the results are shown in Figure S4 and Table 2. The results show that PMIA HF NFMs have stronger hydrophilicity compared to the PMIA substrate by reason that the existence of hydrophilic functional groups, for instance, carboxylic acid and amine terminal groups on the surface of PMIA NFMs [25].

#### 3.2.2. Chemical Properties of the Membrane

To identify the elemental composition of the membrane surface, elemental analysis was implemented on the PMIA HF substrate and PMIA HF NF film surface. XPS was used for the elemental analysis of polymer surfaces. In general, the XPS detection depth was around 5–6 nm, which was much lower than the separation layer thickness [32,34]. Table 3 shows the composition of carbon, oxygen, and nitrogen elements on the PMIA substrates and PMIA HF NFM’s surface.

In general, PA layer structures obtained through the IP process can be classified into three different types according to the O/N ratio. That is, when the calculated O/N ratio is 1.0, the PA structure is fully cross-linked. If the calculated O/N ratio is 2.0, the PA structure is a linear structure substituted by one carboxyl group. Finally, when the calculated O/N ratio is 2.5, it is considered a completely linear structure substituted by two carboxyl groups. Based on the O/N ratio data on the surface of the PMIA NFMs in Table 3, the results in Table 3 show that PMIA NF membranes are more likely to form cross-linked structures, which was similar to the previously reported results [24].

**Figure 6:**
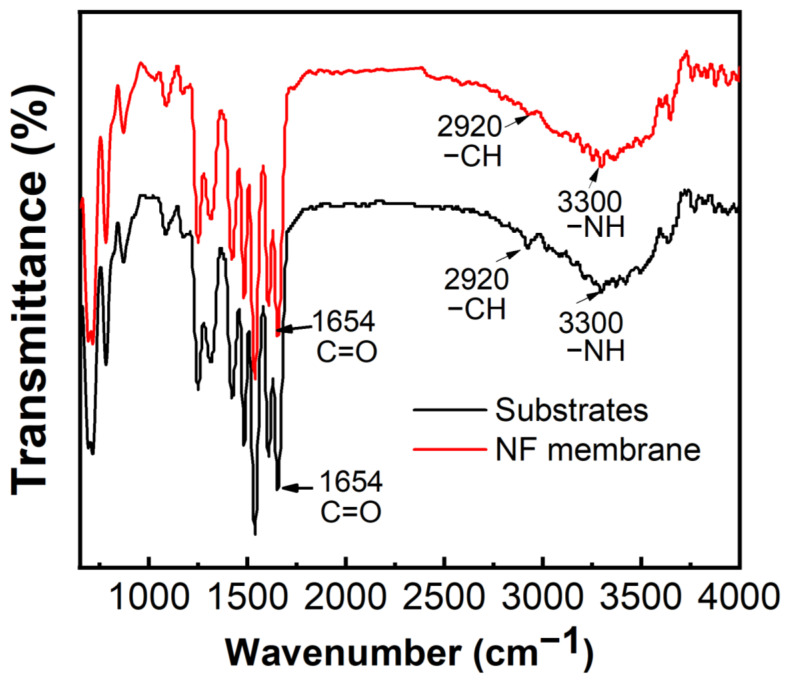
FTIR spectra of PMIA substrate and PMIA TFC membrane.

FTIR was applied to analyze the differences of chemical functional groups on the surface of the PMIA substrate and PMIA HF NFMs, and the results are shown in Figure 6. As can be seen from Figure 6, there is no major change in the spectral peaks between 650~2700 cm^−1^ for the PMIA substrates and PMIA NFMs. This is because both PMIA and PA layers are aromatic polyamide families and therefore have similar functional groups [24]. The peak at 1654 cm^−1^ in the PMIA HF substrate layer and the PA layer is attributed to the C=O asymmetric stretching vibration. Although the PA layer and PMIA HF substrate layer revealed the same peaks at 3300 cm^−1^ (-NH stretching) and peaks around 2920 cm^−1^ (aliphatic C-H bond), while the FTIR intensity at 3300 cm^−1^ and peaks around 2920 cm^−1^ of the PA layer was less than that of the PMIA support layer [35]. This was mainly because the PMIA substrate’s surface was covered with a PA layer, which will affect the FTIR strength of the PMIA NFM’s surface. This is consistent with other studies [23]. All these indicate that the PA layer is triumphantly polymerized on the PMIA HF substrate layer.

### 3.3. Permeate and Salt Retention Performance of PMIA HF TFC NFMs

**Figure 7:**
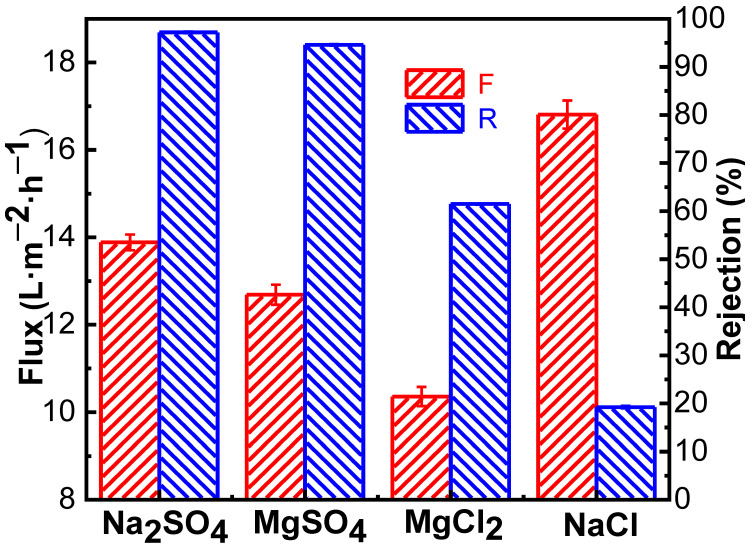
Separation performance of the PMIA HF TFC NF membrane on inorganic salts.

The ionic selectivity of the NF membrane is mainly determined by the combination of the Donnan effect and the screening effect. To investigate the permeate and salt rejection performance of the PMIA HF NFMs on inorganic salts, four typical inorganic salts were selected to investigate the properties of the prepared membrane, and the results are depicted in Figure 7.

As seen in Figure 7, the order of salt retention in different valence states by PMIA hollow fiber nanofiltration membrane is R_Na_2_SO_4__ (98.30%) > R_MgSO_4__ (94.60%) > R_MgCl_2__ (61.48%) > R_NaCl_ (19.24%), which is consistent with the order of retention of salt in different valence states by negative charge NF membrane in the literature [22,27]. This is mainly because Na_2_SO_4_ and NaCl, and MgSO_4_ and MgCl_2_ have the same cation, respectively, and anions with the same charge as the membrane surface were repelled by electrostatic repulsion. The higher the valence state is, the more obvious the repulsion. Thus, the rejection rate of Na_2_SO_4_ was greater than that of NaCl, and that of MgSO_4_ was higher than that of MgCl_2_, which conformed to Donnan’s exclusion effect [24]. As for Na_2_SO_4_ and MgSO_4_ containing the same anion, the hydrated radius of Mg^2+^ (0.428 nm) is higher than the hydrated radius of Na+ (0.358 nm), and the divalent Mg^2+^ was easier to adsorbe on the surface of the negatively charged PMIA HF NFMs, which have adsorption and shielding effects on the surface charge of the membrane [36]. Therefore, when there was the same anion (SO_4_^2−^), the rejection rate of Na_2_SO_4_ was greater than that of MgSO_4_, which was consistent with the results of the compound nanofiltration membrane with a negative charge [37].

**Figure 8:**
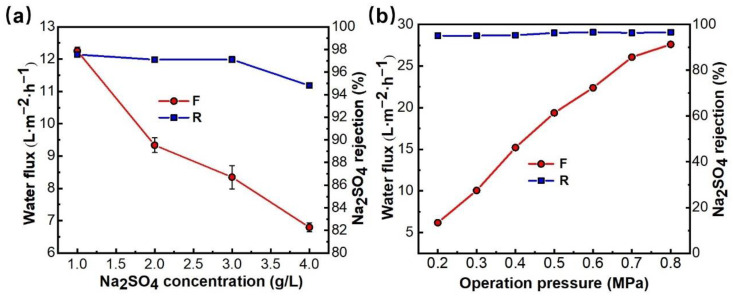
Effects of Na_2_SO_4_ concentration (**a**) and operation pressure (**b**) on the permeability of PMIA.

The separation performance of the PMIA NFMs was different due to the different solution salt concentrations and operation pressure. Therefore, it was of great significance to study the relationship between salt solution concentration and membrane separation performance to understand the membrane separation process. Salt solutions of 1000, 2000, 3000, and 4000 ppm Na_2_SO_4_ were prepared as the solution to be tested, and the influence of solution concentration changes on membrane separation performance were investigated at 25 °C and 0.35 MPa, and the results are displayed in Figure 8a. Figure 8a shows that the rejection rate and flux of the membrane decreased to some extent with the increase of salt concentration of the feed solution. When the operating pressure was constant, the osmotic pressure of the solution increased with the increase of solution salt concentration, and the driving force of the solution through the composite membrane decreased, thereby resulting in a decrease in water flux. The decrease in Na_2_SO_4_ rejection is mainly because PMIA HF TFC NFMs carry a fixed charge, and according to Donnan balance, a higher content of inorganic salts in the feed solution results in a higher concentration of ions of opposite charge in the membrane pore [38]. That is to say, the higher the salt concentration in the membrane pore, the higher the inorganic salt concentration in the osmotic solution, and therefore, the membrane rejection rate is reduced.

Figure 8b shows that the retention rate of Na_2_SO_4_ by the PMIA HF NFMs increased slightly with the increase in operating pressure, and the permeate flux of the NFMs increased in a linear relationship during the pressure increase. The retention rate of the membrane remained stable and the permeate flux of the solution increased from 6.2 to 27.6 L^−1^·m^−2^·h^−1^ when the pressure rose from 0.2 to 0.8 MPa. The effect of operation pressure on the removal of Chromotrope 2B dyes by PMIA HF TFC NF membranes was detailed in Figure S6. The NF test results show that PMIAHF NFMs have excellent pressure resistance, which mainly depends on the excellent mechanical properties of the PMIA substrates (Table S2).

HF NFMs, Test conditions: (a) 0.35 MPa (b) 2000 ppm Na_2_SO_4_, 25 ± 1 °C.

### 3.4. Simulation of Dye Wastewater Treatment

**Figure 9:**
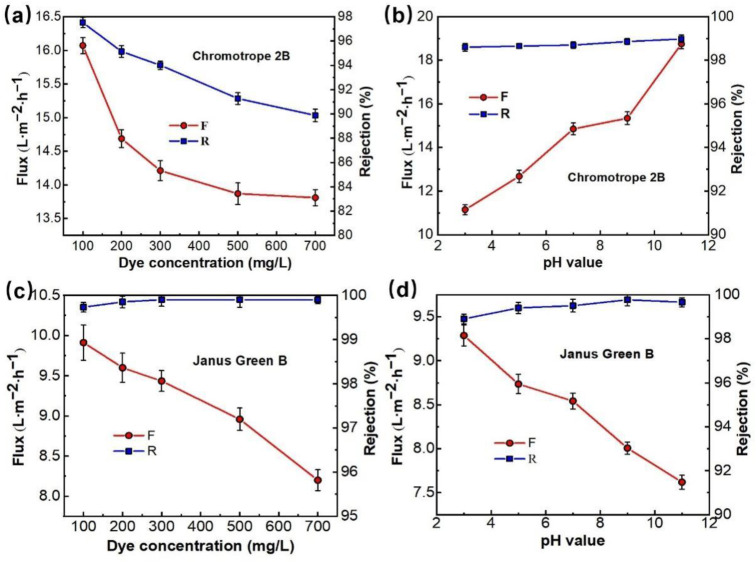
The effect of the dye concentration (**a**,**c**), pH value (**b**,**d**), on separation performance of PMIA HF TFC NF membrane, test conditions: 0.35 MPa, 25 ± 1 °C.

In the actual dye wastewater treatment process, the properties of the membrane vary with the concentration and charge of dye, the pH value, and the inorganic salt concentration in the solution. Two dyes with different charges (chromotrope 2B and Janus Green B) in this study were chosen for the test, the effects of pH and dye concentration on membrane separation performance were investigated, and the results are depicted in Figure 9.

Figure 9a,c shows the effect of dye concentration on the properties of PMIA HF NFMs treating simulated dye wastewater. The dye solutions containing 100, 200, 300, 500, and 700 ppm of chromotrope 2B (513 Da) and Janus Green B (511 Da) were configured to investigate the separation performance of the NFMs with a test pressure of 0.35 MPa. As seen in Figure 9a,c, the membrane flux decreases with increasing concentrations of both chromotrope 2B (513 Da) and Janus Green B (511 Da), mainly because the concentration of the dye in the solution rises, the osmotic pressure of the solution increases, and the concentration polarization in the membrane separation process becomes severe, resulting in a descent in the flux of the membrane as a result of the adsorption of dye molecules on the membrane surface [39]. Under the same dye concentration conditions, the mechanism of flux decrease can be analyzed by comparing the flux magnitude of the two longitudinally. Chromotrope 2B has the same charge as the surface of the nanofiltration membrane, which has a mutual repulsive effect and does not easily adsorb on the membrane surface, saturating the membrane surface after 500 ppm, and slowly decreasing flux at 700 ppm. In contrast, Janus Green B has the opposite charge to the membrane surface and the charges attract each other, so the dye is easily adsorbed on the NMF’s surface and clogs the membrane pores, resulting in a linear decrease in membrane flux as the dye concentration increases. At the same time, Figure 9a,c shows that when the dye concentration increased from 100 ppm to 700 ppm, the rejection rate of chromotrope 2B reduced from 97.5% to 89.9%, while the retention rate of the positively charged Janus Green B dye increased from 99.7% to 99.9%, reflecting that the dye molecules are easily adsorbed on the membrane surface when it is in contact with the surface of the membrane. The retention rate increased because the pores of the membrane were clogged and the pore size became smaller.

Figure 9b,d shows the effect of pH values on the properties of PMIA HF NFMs treating simulated dye wastewater. Comparative experiments were conducted by configuring a concentration of 100 ppm chromotrope 2B (negatively charged) and Janus Green B (positively charged) dye solutions, respectively, with a test pressure of 0.35 MPa and a pH range of 3 to 11, and the results are represented in Figure 9b,d.

Figure 9b shows that the change in the solution pH value had a great influence on the fluxes of chromotrope 2B dye solutions. This is mainly because chromotrope 2B was an acid dye. In industrial production, when used for acid dye, the optimal pH range for dyeing is 2.5~4. Therefore, when pH = 3, the negative charge on the membrane surface becomes weaker, which is more beneficial to the adsorption and deposition of negatively charged chromotrope 2B molecules on the membrane surface to form a filter cake layer, thus leading to the lowest achieved flux [40]. With the increase of the solution pH value, the negative charge on the membrane surface increases. Under the action of electrostatic repulsion on the membrane surface, the adsorption ability of negatively charged chromotrope 2B dye molecules on the membrane surface decreased [41], so the flux of the PMIA NFMs increased with the rise in pH value. On the contrary, for the positively charged Janus Green B dye, the flux of the membrane decreases with increasing pH, which also verifies this statement.

Meanwhile, Figure 9b,d show that the change of pH value does not have much effect on the retention rate of both dyes, and the rejection rate of the PMIA HF NFMs retains more than 98% of both dyes in the pH value range from 3 to 11, which is mainly because of the large molecular weight and molecular size of the dyes, and the sieving effect plays a major role, so the retention rate of the membrane remains relatively stable for both dyes. The retention rate of Janus Green B is above 99%, which is partly due to the sieving effect, but may also be due to the positively charged Janus Green B dye being adsorbed on the membrane surface to form a filter cake layer and the membrane pores becoming smaller, resulting in a higher retention rate.

**Figure 10:**
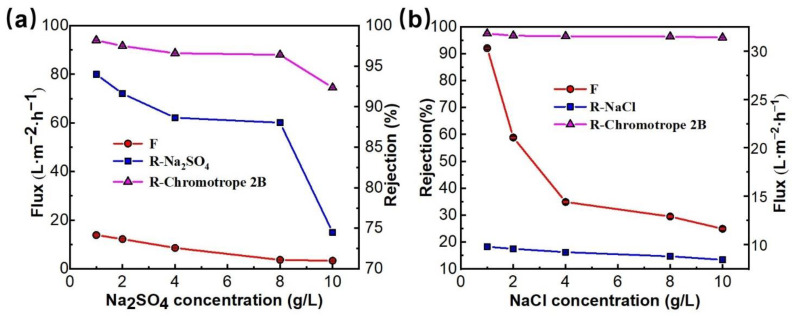
The effect of the inorganic salt (Na_2_SO_4_ (**a**), NaCl (**b**)) concentration on separation performance of PMIA HF TFC NF membrane, test conditions: 0.35 MPa, 25 ± 1 °C.

Figure 10a,b showed the effect of Na_2_SO_4_ and NaCl concentrations on the dye removal performance of PMIA HF NFMs. As seen in Figure 10a,b, the flux of the NFMs showed a decreased trend as the concentration of salts rose from 1 g/L to 10 g/L. This was mainly because, with the increase of the concentration of salt in the solution, the osmotic pressure of the solution increases, leading to the reduction of the driving forces on both sides of the membrane, thus reducing the flux [42]. Additionally, an increase in the salt concentration of the solution makes the concentration of Na^+^ in the solution increase. This positive action has a shielding effect on the surface of the negatively charged membrane, thereby making the dye molecules easy to be adsorbed on the surface of the membrane, which consequently causes the blockage of the membrane holes, leading to a decline in the membrane flux.

When the inorganic salt was Na_2_SO_4_, the rejection of the membrane to chromotrope 2B decreased from 97.5 to 74.7% with the rise in salt concentration (0 g/L to 10 g/L). When the inorganic salt was NaCl, the dye rejection rate decreased from 97.5 to 96.0%. This is because the salt ions in the mixing system will be coupled with charged chromotrope 2B, which makes the dye disperse more evenly. Additionally, it was easier for it to pass through the membrane holes, thus leading to the reduction in the rejection rate of chromotrope 2B. In addition, the rejection rate of Na_2_SO_4_ also descended with a rise in salt concentration, especially when the salt concentration was more than 8 g/L, and the membrane rejection rate decreased significantly from 88.3% to 74.5%. Because the increase in salt concentration also causes an increase in the concentration gradient on the membrane surface, and the increase in the cation Na^+^ in the solution system has a certain shielding effect on the NF membrane surface (the electrostatic repulsion between the negatively charged membrane surface and SO_4_^2−^ ions is weakened) [43], which increases the solute flux to the membrane and leads to a falloff in the rejection rate of the membrane for salt, while the concentration of NaCl in the solution has less effect on the dye rejection rate than Na_2_SO_4_, mainly because both Na^+^ and Cl^−^ ions have the same monovalence and both have a small charge-shielding effect [40]. Therefore, the effect on the dye rejection rate is also small.

### 3.5. Continuous Stability Operation of the Membrane

**Figure 11:**
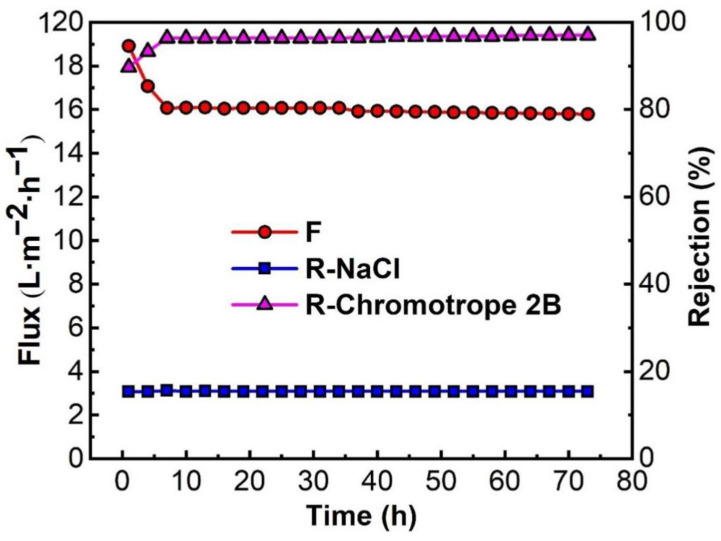
Continuous stable operation test of the PMIA HF NF membrane. Test conditions: 100 ppm chromotrope 2B and 4000 ppm NaCl, 0.35 MPa, 25 ± 1 °C.

Continuous stable operation is one of the most important indexes to investigate the comprehensive performance of PMIA HF TFC NFMs [44]. To investigate the continuous stable operation of the membrane desalination, the mixed solution of 100 ppm chromotrope 2B and 4000 ppm NaCl was configured to conduct continuous stable operation experiments for 72 h at 0.35 MPa and 25 °C. The experimental data were recorded every 3 h and the experimental results are depicted in Figure 11, and the effect of operation time on the removal of Chromotrope 2B dyes by PMIA HF NFMs was displayed in Figure S6. Figure 11 shows that the membrane flux decreased significantly in the first 7 h, mainly because some chromotrope 2B dye molecules were adsorbed on the surface of the membrane to build a filter cake layer, and the membrane was compacted under pressure. When the time exceeded 7 h, the membrane flux almost reached a stable state, and the membrane retention rate of dye was more than 96.0%, while the rejection rate of NaCl remained at about 15%. Comprehensive analysis showed that the membrane had a high rejection effect on dye in the desalination test of chromotrope 2B dye, and a high pass rate on inorganic salt NaCl, it shows that the membrane has good potential for desalination of dye wastewater.

## 4. Conclusions

PMIA HF TFC NFMs were successfully fabricated by IP technology with a self-made PMIA HF substrate. The effects of preparation conditions on the properties of the membrane were systematically investigated. Under the optimum preparation conditions, the PWF of the PMIA HF NFMs was 18.2 L·m^−2^·h^−1^ (0.35 MPa), and the rejection rate of Na_2_SO_4_ was 98.3%. The characteristics of the HF TFC NFMs’ surface were observed and analyzed by SEM, AFM, XPS, and FTIR spectra. It was confirmed that a layer of PA separation layer was developed on the surface of the PMIA substrates, which was composed of an ultra-thin dense layer and spherical structure on the surface of the dense layer. PMIA HF TFC NFMs were applied to the treatment of printing and dyeing wastewater and they showed excellent pressure resistance, separation, and flux stability.

## Figures and Tables

**Table 1:** Characteristics of the Chromotrope 2B and Janus Green B.

Dye Name	Molecular Structure	Dye Types	Relative Molecular Weight (Da)	Charge
Chromotrope 2B	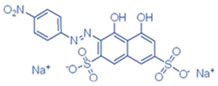	Acid dyes	513.37	−2
Janus Green B	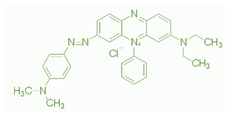	Basic dyes	511.06	+1

**Table 2:** Surface properties of membranes.

Membrane	Roughness Values		Water Contact Angle (^o^)
	Ra	Rq	
PMIA substrate	15 ± 0.32	19 ± 0.31	65.3 ± 2.7
PMIA TFC NF	43 ± 0.43	58 ± 0.46	54.6 ± 3.3

**Table 3:** XPS analysis membrane samples.

Samples	Atomic Composition from XPS (%)			
	C/(%)	O/(%)	N/(%)	O/N
PMIA substrate	72.26	17.44	10.3	1.69
PMIA TFC NF membrane	73.41	15.33	11.26	1.36
Theoretically calculated date Totally crosslinking structure	71.42	14.29	14.29	1.0
Fully linear structure substituted with two carboxyl	65.00	25.00	10.00	2.50
Linear structure substituted with one carboxyl	68.42	21.05	10.53	2.00

## Data Availability

The data presented in this study are available on request from the corresponding author.

## Supplementary Materials

The following supporting information can be downloaded at: https://www.mdpi.com/article/10.3390/membranes12121258/s1, Figure S1: The casting equipment of the PMIA HF substrates; Figure S2: Pictures of PMIA HF substrate and SEM images of its cross-section at different magnifications; Figure S3: The pore size and the pore size distribution of PMIA HF substrate; Figure S4: The contact angle of PMIA HF substrate and PMIA HF NF membrane; Figure S5: The rejection of PEGs with different molecular weights through PMIA HF TFC NF membrane; Figure S6: Effect of operation pressure on the removal of Chromotrope 2B dyes by PMIA HF TFC NF membranes; Figure S7: Effect of operation time on the removal of Chromotrope 2B dyes by PMIA HF TFC NF membranes; Table S1: The properties of PMIA HF substrate; Table S2: Mechanical properties of PMIA HF substrates.
