# Supplementary material for: Optimization of Preparation Conditions of Poly(m-phenylene isophthalamide) PMIA Hollow Fiber Nanofiltration Membranes for Dye/Salt Wastewater Treatment
Source: Membranes (Basel). 2022 Dec 13;12(12):1258. doi: 10.3390/membranes12121258 (PMC9783120; doi:10.3390/membranes12121258)
Supplement: Supplementary file 1 [file membranes-12-01258-s001.zip › membranes-2074537-supplementary.pdf]

Supporting information

Additional information

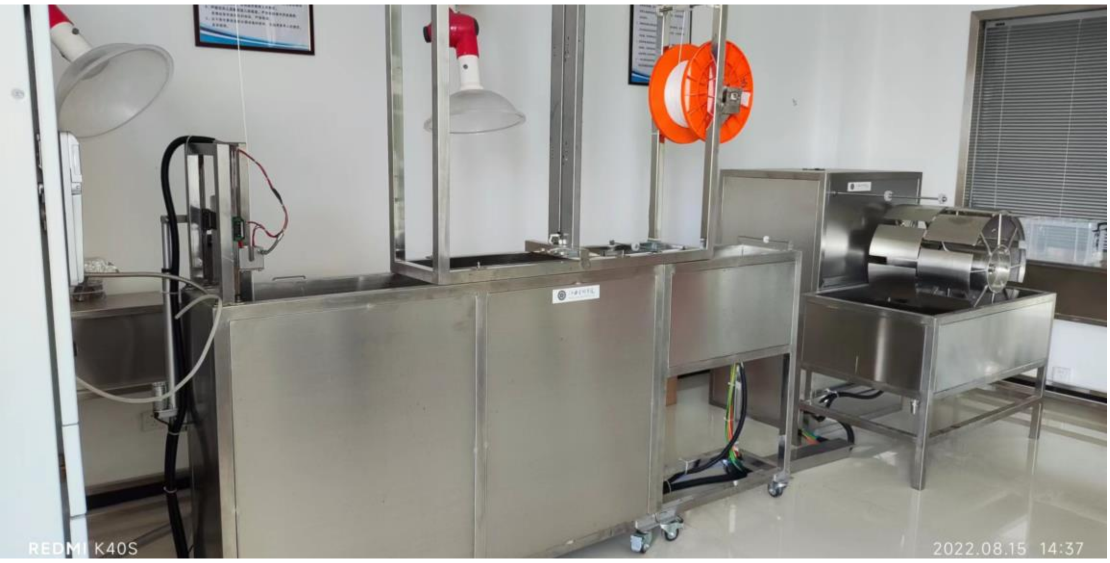

Figure S1. The casting equipment of the PMIA HF substrates.

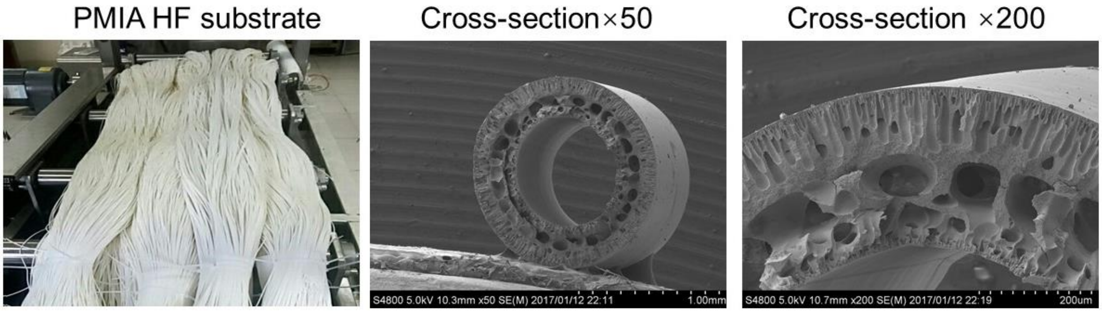

Figure S2. Pictures of PMIA HF substrate and SEM images of its cross-section.
at different magnifications

The cross-sectional morphology of the PMIA substrate is shown in Fig.S2. As can be seen in Fig.S2, a typical asymmetric structure can be observed on the PMIA substrate, which usually has a dense skin layer, finger pores, sponge layer, and large cavity structure. The large pore structure facilitates the permeability of the membrane.

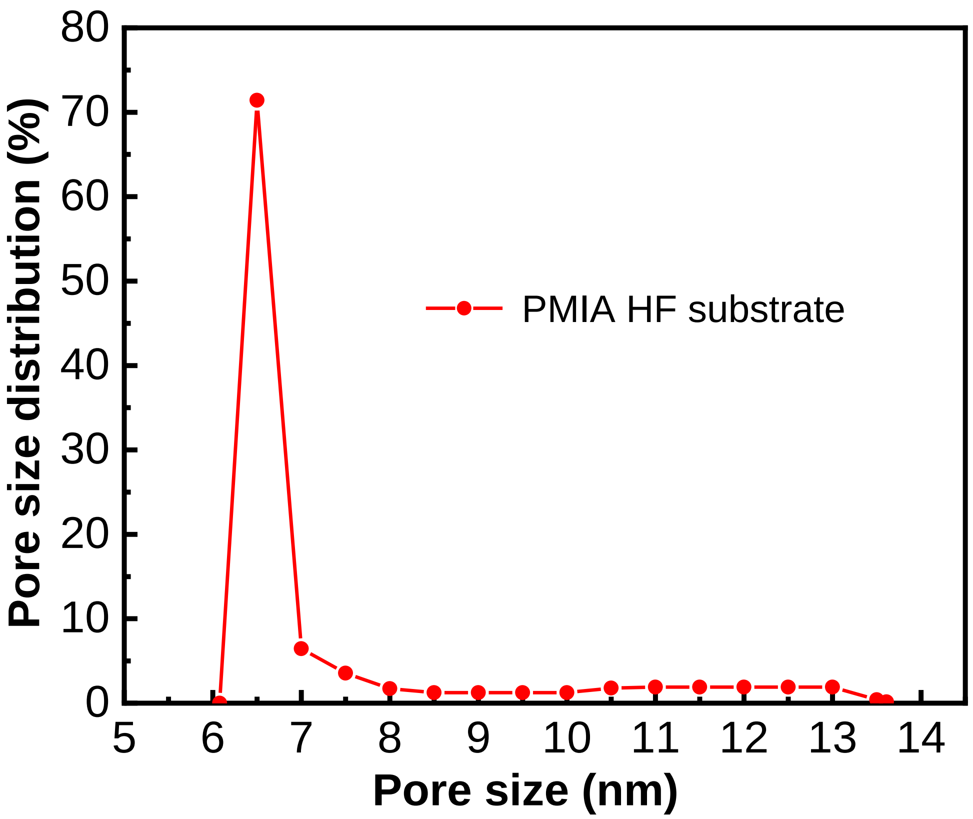

Figure S3. The pore size and the pore size distribution of PMIA HF substrate.

The pore size and pore size distribution of PMIA HF substrate are shown in Fig. S3, fit can be seen from Fig.S3, that the pore size distribution of PMIA substrate membrane is uniform, mainly concentrated between 6-7 nm, with an average pore size of 6.5 nm. it is known that uniform pore size of substrate membrane is more favorable to the distribution of interfacially polymerized polyamide separation layer.

Table S1. The properties of PMIA HF substrate.

Substrate	Outer diameter (mm)	Inner diameter (mm)	Thickness (mm)	Average pore size (nm)	Pure water flux ($L\cdot m^{-2}\cdot h^{-1}\cdot bar^{-1}$)	BSA Rejection (1000 ppm) (%)
PMIA	1.2	0.65	0.28	6.5	159.5	97.3

Table S2. Mechanical properties of PMIA HF substrates.

Substrate	Tensile strength (MPa）	Breaking elongation (%）	Yang's modulus(MPa）
PMIA	3.8 ± 0.1	63.0 ± 3.4	162.8 ± 2.1

Mechanical properties of PMIA high-frequency substrate are shown in Table S2, it can be seen From Table S2 that PMIA HF substrate has excellent mechanical properties, it is known that the substrate mainly plays a supporting role in the operation of nanofiltration membrane, and the better mechanical properties of the substrate means the better resistance of the prepared nanofiltration membrane. This is a prerequisite for the excellent pressure resistance of PMIA HF TFC nanofiltration membranes.

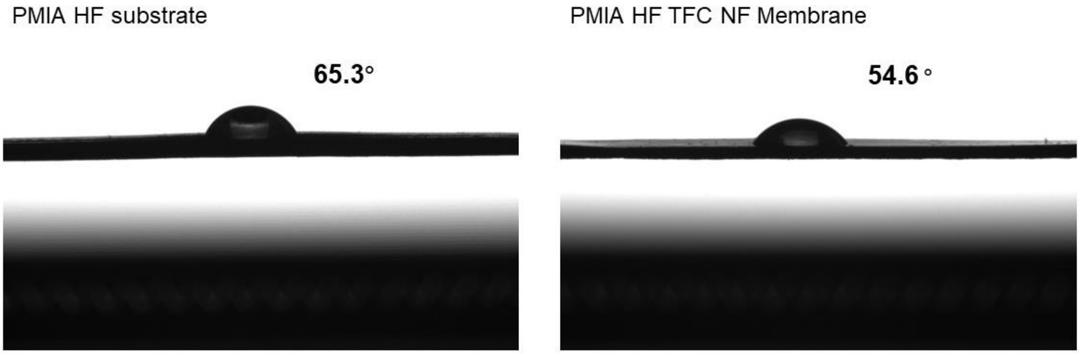

Figure S4. The contact angle of PMIA HF substrate and PMIA HF NF membrane.

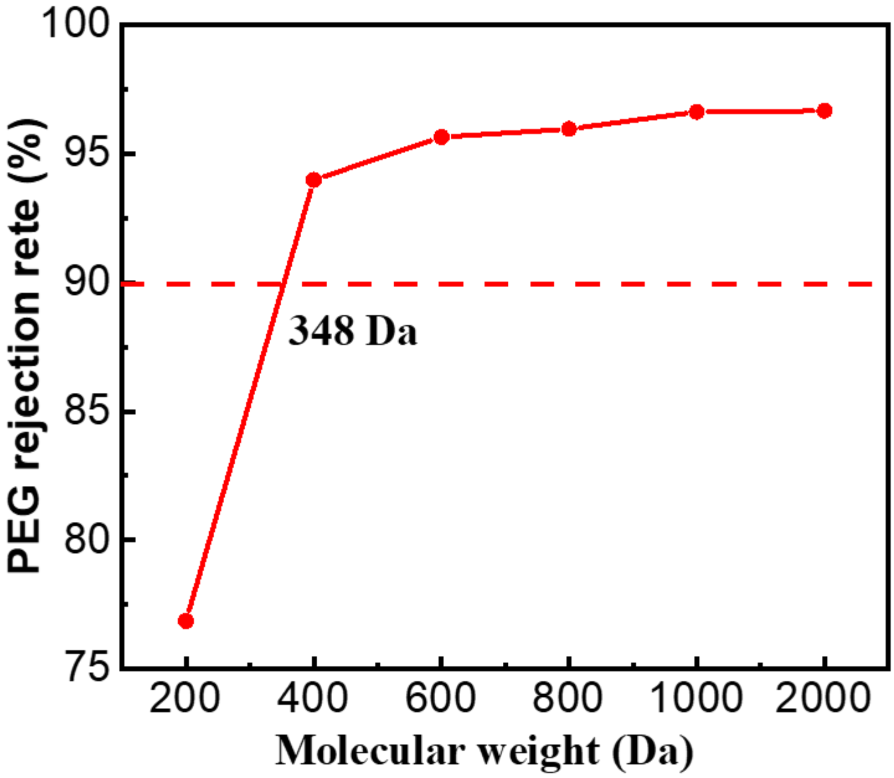

Figure S5. The rejection of PEGs with different molecular weights through PMIA HF TFC NF membrane.

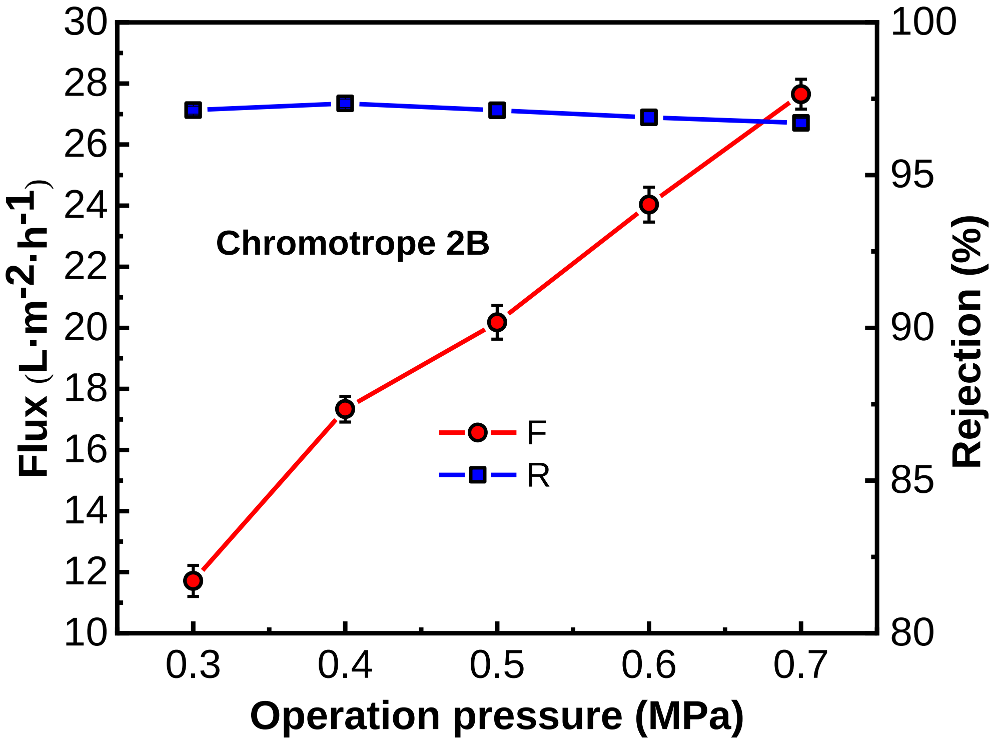

Figure S6. Effect of operation pressure on the removal of Chromotrope 2B dyes by PMIA HF TFC NF membranes Test conditions:100 ppm chromotrope 2B, 25 ± 1°C.

A 100 ppm aqueous solution of Chromotrope 2B was configured and the experiment was run continuously for 10 h at 0.35 MPa and room temperature, and the experimental results are shown in Fig.S6. As can be seen from Fig.S7, the analysis of the test results revealed that the permeate flux of the PMIA HF NF membrane decreased significantly in the early stage, which was due to the adsorption of some dye molecules on the membrane surface to form a filter cake layer. With the increase of the test time, the permeate flux gradually stabilized until about 2h of operation but still had a slowly decreasing trend, and the retention rate of dye by the membrane was always close to 98.0% during the whole process. Comprehensive analysis shows that the hollow fiber nanofiltration membrane has stable separation performance for dye discoloration acid 2B.

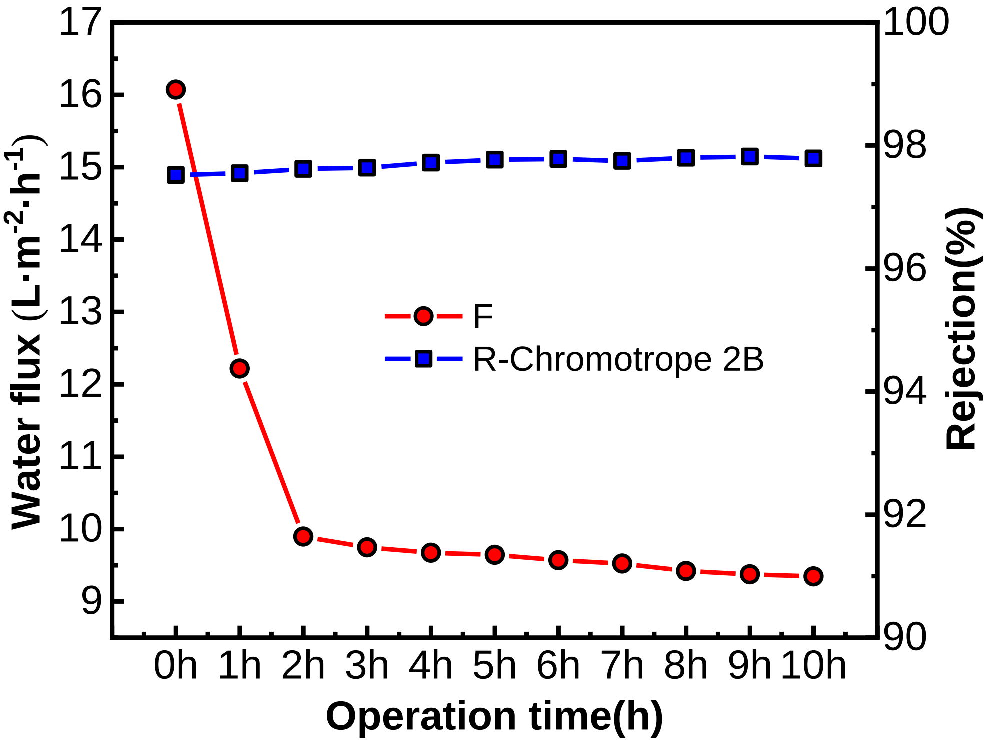

Figure S7. Effect of operation time on the removal of Chromotrope 2B dyes by PMIA HF TFC NF membranes Test conditions:100 ppm chromotrope 2B, 0.35 MPa, 25 ± 1℃.
